# Characterization of Foot-and-Mouth Disease Viruses in Zambia-Implications for the Epidemiology of the Disease in Southern Africa

**DOI:** 10.3390/v13112195

**Published:** 2021-10-31

**Authors:** Frank Banda, Yona Sinkala, Liywalli Mataa, Phiyani Lebea, Tingiya Sikombe, Henry L. Kangwa, Elliot M. Fana, Mokganedi Mokopasetso, Jemma Wadsworth, Nick J. Knowles, Donald P. King, Melvyn Quan

**Affiliations:** 1 Central Veterinary Research Institute, Lusaka 10101, Zambia; tingiyasikombe@gmail.com (T.S.); henrylombekangwa@gmail.com (H.L.K.); 2 Department of Veterinary Tropical Diseases, Faculty of Veterinary Science, University of Pretoria, Pretoria 0002, South Africa; melvyn.quan@up.ac.za; 3 Department of Veterinary Services, Ministry of Fisheries and Livestock, Lusaka 10101, Zambia; ysinkala@gmail.com (Y.S.); lmataa_nmataa@yahoo.com (L.M.); 4 Tokabio (Pty) Limited, Pretoria 0001, South Africa; phiyani@gmail.com; 5 Botswana Vaccine Institute, Lejara, Gaborone 5617, Botswana; efana@bvi.co.bw (E.M.F.); mmokopasetso@bvi.co.bw (M.M.); 6 The Pirbright Institute, Pirbright GU24 0NF, UK; jemma.wadsworth@pirbright.ac.uk (J.W.); nick.knowles@pirbright.ac.uk (N.J.K.); donald.king@pirbright.ac.uk (D.P.K.)

**Keywords:** FMD, livestock, buffalo

## Abstract

The livestock industry supports livelihood and nutritional security of at least 42% of people in the Southern African Development Community region. However, presence of animal diseases such as foot-and-mouth disease poses a major threat to the development of this industry. Samples collected from FMD outbreaks in Zambia during 2015–2020, comprising epithelial tissues samples (*n* = 47) and sera (*n* = 120), were analysed. FMD virus was serotyped in 26 samples, while 92 sera samples tested positive on NSP-ELISA. Phylogenetic analysis revealed notable changes in the epidemiology of FMD in Zambia, which included: (i) introduction of a novel FMDV SAT-3 (topotype II) causing FMD cases in cattle in Western Province; (ii) emergence of FMDV serotype O (topotype O/EA-2) in Central, Southern, Copperbelt, Western, Lusaka Provinces; and (iii) new outbreaks due to SAT -2 (topotypes I) in Eastern Zambia. Together, these data describe eight different epizootics that occurred in Zambia, four of which were outside the known FMD high-risk areas. This study highlights the complex epidemiology of FMD in Zambia, where the country represents an interface between East Africa (Pool 4) and Southern Africa (Pool 6). These changing viral dynamics have direct impacts on FMD vaccine selection in the SADC region.

## 1. Introduction

Foot-and-mouth disease (FMD) is a highly contagious transboundary animal disease (TAD) that is a major concern to livestock industries throughout the world. The disease is caused by the FMD virus (FMDV), which affects cloven-hoofed animals. In addition to the ability of FMDV to spread rapidly within domesticated livestock, the virus can be maintained within populations of certain wildlife species, such as free-ranging African buffalo (*Syncerus caffer*), which do not show obvious signs of disease and can act as virus reservoirs capable of transmitting the virus to livestock [1,2,3]. The involvement of wildlife in the epidemiology of FMD provides a challenge to control FMD in sub-Saharan Africa [4].

The FMDV genome contains a single open reading frame that encodes four structural viral proteins (VP1–VP4) that form the virus capsid, and eight non-structural proteins (NSP) involved in the replication of the virus. VP1 sequences are often used for epidemiological investigations [5] to characterise FMD viruses within the seven serotypes (A, O, C, SAT-1, SAT-2, SAT-3 and Asia-1) and into genetically discrete topotypes. Globally, there are seven endemic pools of infection which often require tailored diagnostics and vaccines [6,7]. In Africa, five serotypes (O, A, SAT-1, SAT-2, SAT-3) are present, but their distribution varies. In Southern Africa (Pool 6), three serotypes (SAT 1-3) are endemic with sporadic incursions of serotype O and A recorded in Northern Zambia as a consequence of spillover from Pool 4 (East Africa) [8,9,10]. The distribution of these diverse serotypes and subtypes of FMDV is subject to change, and requires continuous monitoring [10,11].

FMD continues to be a major sanitary concern for beef producers in the Southern African Development Community (SADC) region because the disease is a key constraint to access high-value export markets. The occurrence of FMD results in damaging consequences for the livelihoods of local farmers due to the disease’s impacts on productivity, food security and loss of income [12]. Furthermore, in Zambia, FMD impedes the utilisation of livestock and wildlife resources of the country, where 80% of the population rely on agriculture for food and income generation [13]. In this context, FMD in the country has been likened to a disease of poverty because of its negative effects, and is the reason for low economic growth [14,15].

**Figure 1:**
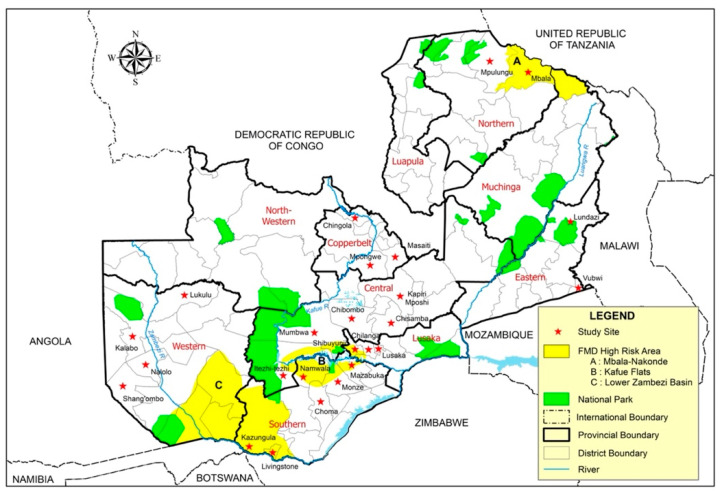
Foot-and-mouth disease high-risk areas and study sites in Zambia.

In Zambia, FMD was first reported in 1933 [16], and sporadic outbreaks of the disease have been reported from three high-risk areas [14,17]. These defined high-risk areas (Figure 1) are (A) the Northern part of the country on the border with Tanzania (Nakonde, Mbala and Mpulungu districts), (B) the Kafue Flats on the border between Central and Southern provinces (Namwala, Mumbwa, Itezhi tezhi and parts of Monze, Choma and Mazabuka districts) and (C) the Southern parts of Western and Southern Provinces (from Kazungula/Livingstone to a point beyond Sesheke district, where the border with Angola turns to the north-west). An unprecedented rise in the incidence of FMD outbreaks in livestock since 2004 has been observed in Southern Africa [18,19]. The reasons for this are most likely multifactorial, but prima facie evidence points to the poor performance of prophylactic vaccination programmes [20]. In Zambia, a rise in FMD reports was recorded in Western, Eastern, Southern and Central Provinces of the country from 2015 to 2020. The main risk factors have been reported elsewhere [21,22], while the molecular epidemiology of FMD in the country has not been fully elucidated. Previous FMD outbreaks in Zambia have been difficult to prevent and control [17], and have not been systematically studied to gain insight into outbreak causation or transmission patterns, and also to predict when the next outbreak would likely occur [22]. This information is necessary so that appropriate preventive measures can be instituted. This study aimed to characterize FMD viruses causing recent cases in Zambia, in order to provide data to help understand the epidemiology of the disease in Southern Africa.

## 2. Materials and Methods

### 2.1. Study Area

The study region encompassed the whole of Zambia, where specific sampling areas were purposely selected based on suspected FMD cases reported to the Department of Veterinary Services during the study period of 2015 to 2020.

### 2.2. Sample Collection 

A total of 75 kraals and farms were visited during FMD outbreak investigations. After clinical examination for the presence of typical FMD lesions, fresh epithelial tissue, vesicular fluid and blood samples from suspect FMD cases in these outbreak areas were collected. The tissue specimens and blood samples were collected, as described elsewhere [23]. Briefly, at least one gram of epithelial tissue was collected from an unruptured or recently ruptured vesicle and placed in a transport medium composed of equal amounts of glycerol and 0.04 M phosphate buffer (pH 7.2–7.6). Plain vacutainer tubes (Becton, Dickson and Company, Franklin Lakes, NJ, USA) were used to collect whole blood, and the serum was separated once the blood was clotted. Epithelial tissues and sera samples were kept refrigerated at 4 °C, or on ice for epithelial tissue until received at the Central Veterinary Research Institute (CVRI) in Zambia, where samples were stored at −20 °C. The samples were packaged and shipped to International Reference Laboratories (World Organisation for Animal Health (OIE) Reference Laboratory, Botswana Vaccine Institute (BVI) and the Food and Agriculture Organization (FAO) World Reference Laboratory for FMD (WRLFMD) and OIE Reference Laboratory, The Pirbright Institute, United Kingdom) for virus isolation, NSP-ELISA, Antigen-ELISA, real-time reverse transcription PCR (RT-qPCR) and VP1 sequencing. During the study period, 35 samples were submitted to BVI and 13 samples were submitted to WRLFMD.

### 2.3. Laboratory Analysis of Samples

At CVRI, all sera samples were tested for the presence of FMDV NSP-specific antibodies using a multi-species antibody test kit (IDEXX, Westbrook, ME, USA) by following the manufacturer’s instructions. Representative epithelial samples were prepared and tested for the presence of FMDV by thawing and blot drying them, followed by grinding in phosphate-buffered saline (PBS) using sterile sand in a pestle and mortar in a 10% suspension [24]. The suspensions were tested using a sandwich ELISA (IZSLER Biotechnology Laboratory, Brescia, Italy) for detection and serotyping of FMDV antigens by following the manufacturer’s instructions. At the WRLFMD, virus isolation was carried out on primary bovine thyroid (BTy) cells [25] or IB-RS-2 cell cultures [26], and an Ag-ELISA was performed on cell cultures showing cytopathic effect to identify the serotype [24,27,28]. At the BVI, lamb kidney cells were used for virus isolation. FMDV-specific RT-qPCR assays were performed on the samples [29,30]. Samples with detectable FMDV genomes were further characterised by VP1 sequencing [31]. Complete VP1 nucleotide sequences were aligned using BioEdit 7.0.5.3 [32,33]. Optimal nucleotide substitution models were computed for each serotype using MEGA 7 [34]. The maximum likelihood algorithm was used to construct phylogenetic trees employing MEGA 7. One thousand bootstrap pseudo-replicates were used to assess branching confidence.

## 3. Results

### 3.1. Epidemiology and Clinical Observations

**Figure 2:**
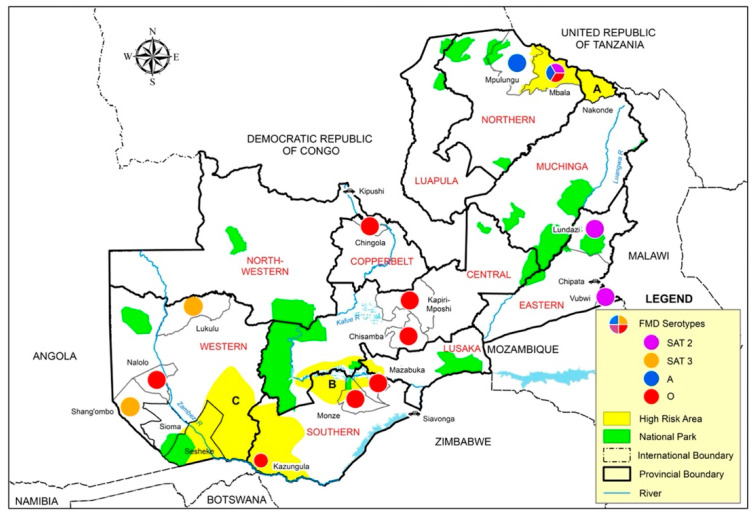
Foot-and-mouth disease virus serotypes detected in Zambia between 2015 and 2020.

Twenty-five FMD outbreaks were recorded during the study period, of which 32% (8) were primary outbreaks and 68% (17) were outbreak extensions (Table 1 and Figure 2). Out of the eight primary outbreaks, four (50%) were reported outside the three known high-risk areas in Zambia (Figure 2): in October 2015 from Shang’ombo district of Western Zambia; in March 2018 and January 2019 in Chisamba district of Central Zambia and finally during March 2019 in Lundazi and Vubwi districts of Eastern Zambia (Figure 2). The other four primary outbreaks were reported in February 2015 from Mpulungu and Mbala districts of Northern Zambia, whilst Kawimbe Veterinary Camp of Mbala district reported outbreaks in October and March 2018.

Affected cattle showed typical clinical signs of FMD, which included drooling, nasal discharge, the grinding of teeth, and mouth lesions on the tongue, dental pad and gums. Most animals recovered within two to three weeks. Vesicles were observed as a whitish area that ruptured when pressure was applied, and erosions were observed on the feet. Vesicles were also apparent on the mammary glands in some of the dairy animals, and one instance sloughing of the teat was observed. Pathognomonic clinical signs of FMD were observed in pigs and piglets at Hamangaba and Makeni veterinary camps in Monze and Chilanga districts, respectively. Animals were seen with white lesions and blisters on the coronary bands of the hoof and snout. There was a loss of appetite, and the piglets showed mild lameness with a reluctance to move. Severe lesions of the heel pad area were observed. In Monze, the horn of the hoof in one piglet separated and shed, as has been observed elsewhere [1].

Out of the blood samples collected, 110 (92%) were bovine samples and 10 (8%) were swine samples. A total of 92 (77%) were positive for antibodies against FMDV NSPs with a percentage inhibition range of 60.8 to 95.9% (Table 1). Despite testing positive with the RT-qPCR assay, samples collected from Chisamba 2018 and Itezhi Tezhi were negative when tested with the NSP-ELISA at initial sampling, but subsequent samples collected after 14 days were positive, probably due to the lack of NSP antibodies during the early stages of disease at the time of initial collection. All pig samples tested positive with the NSP-ELISA, and were typed as serotype O with the Ag-ELISA. Samples collected from Kazungula district Nyawa camp also tested positive with the RT-qPCR assay, and were typed as serotype O with the Ag-ELISA. Twenty-six samples contained detectable FMDV genomes and could be serotyped, while no virus genome was detected in the remainder of the samples. FMDV positive samples were further characterised by VP1 sequencing.

### 3.2. Phylogenetic Analyses

The most appropriate nucleotide substitution models for each serotype were found to be the Tamura-Nei (TN-93) model, gamma-distributed with invariant sites (G+I) for type O; the Hasegawa-Kishino-Yano (HKY) model, G+I, for types A and SAT2; and the General Time Reversible (GTR) model, G+I, for serotype SAT-3.

**Figure 3:**
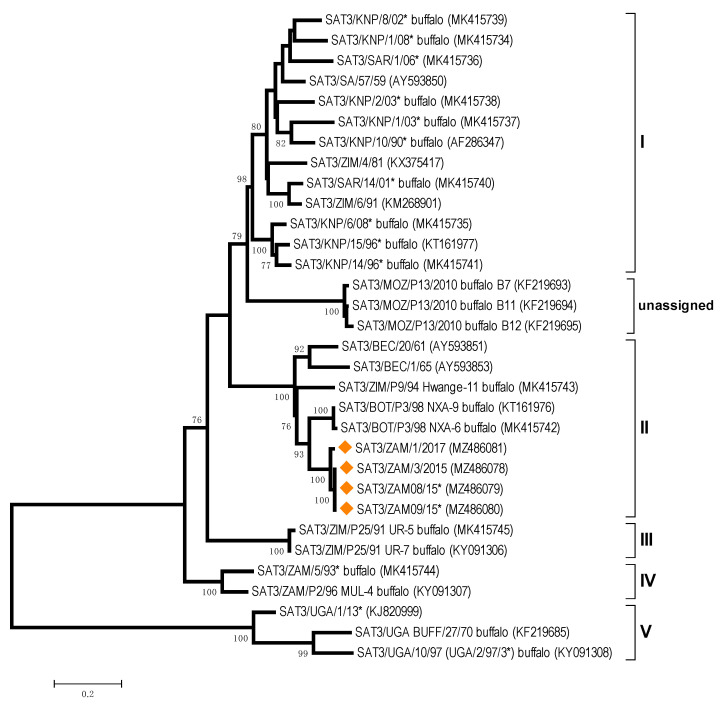
Midpoint-rooted maximum likelihood tree showing the relationships between the VP1 sequences of the 2015 foot-and-mouth disease virus SAT-3 serotypes from Zambia (indicated with diamonds) and other contemporary and reference viruses. * Reference number not assigned by the WRLFMD.

The study revealed that FMDV SAT-3 was responsible for the outbreak reported in Beshe Vet Camp in Shangombo district of Western Province in 2015 (isolate ZAM/3/2015 and two additional sequences for isolates from BVI). Further outbreaks linked to these cases were later reported in all districts of Western Province except for Mwandi district [35]. Sequence data supported this close relationship, where nucleotide (nt) identities between ZAM/3/2015 and ZAM/1/2017 were 98.0%. Beyond the genetic identity between Zambia FMDV sequences, ZAM/3/2015 was most closely related to SAT3/BOT/P3/98 NXA-9 (88.6% nt identity) and SAT3/BOT/P3/98 NXA-6 (88.3% nt identity) (Figure 3).

**Figure 4:**
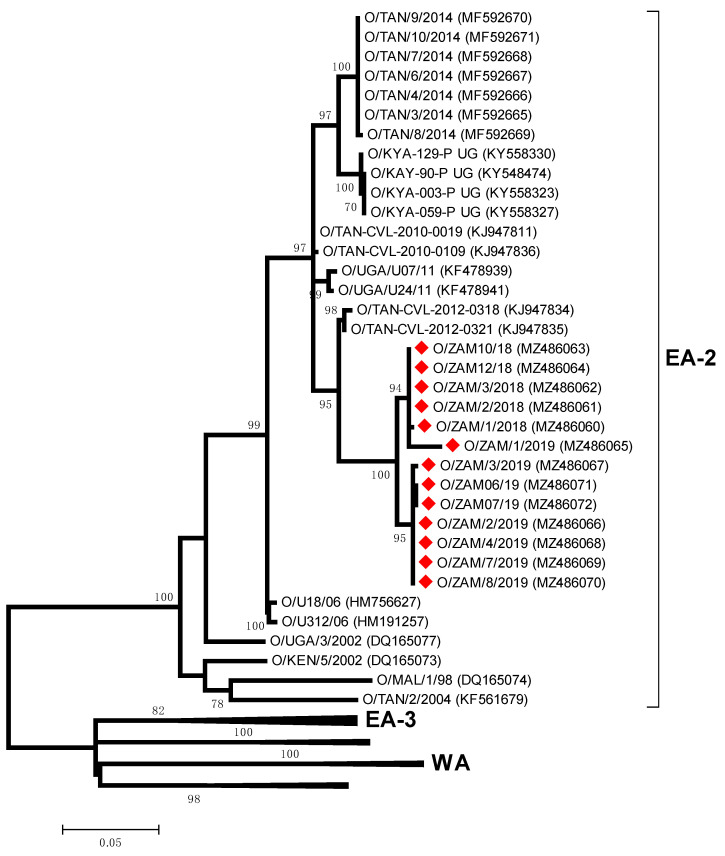
Midpoint-rooted maximum likelihood tree showing the relationships between the VP1 sequences of the 2018 foot-and-mouth disease virus O serotypes from Zambia (indicated with diamonds) and other contemporary and reference viruses.

All the serotype O viruses isolated in this study (Table 1) clustered within the East Africa 2 (EA-2) topotype, most closely related to viruses collected in Tanzania: O/TAN-CVL-2012-0318 (95.8–95.9% nt identity) and O/TAN-CVL-2012-0321 (95.9–96.1% nt identity) (Figure 4). These included FMD samples collected from six provinces in Zambia (Central, Copperbelt, Lusaka, Northern, Southern, Western Provinces). Phylogenetic analysis of O/ZAM/1/2018 from Chisamba, Central Zambia and O/ZAM/10/2018 from Kawimbe, Mbala Northern Zambia revealed that the viruses responsible for these two primary outbreaks were closely related to each other (99.8% nt identity).

**Figure 5:**
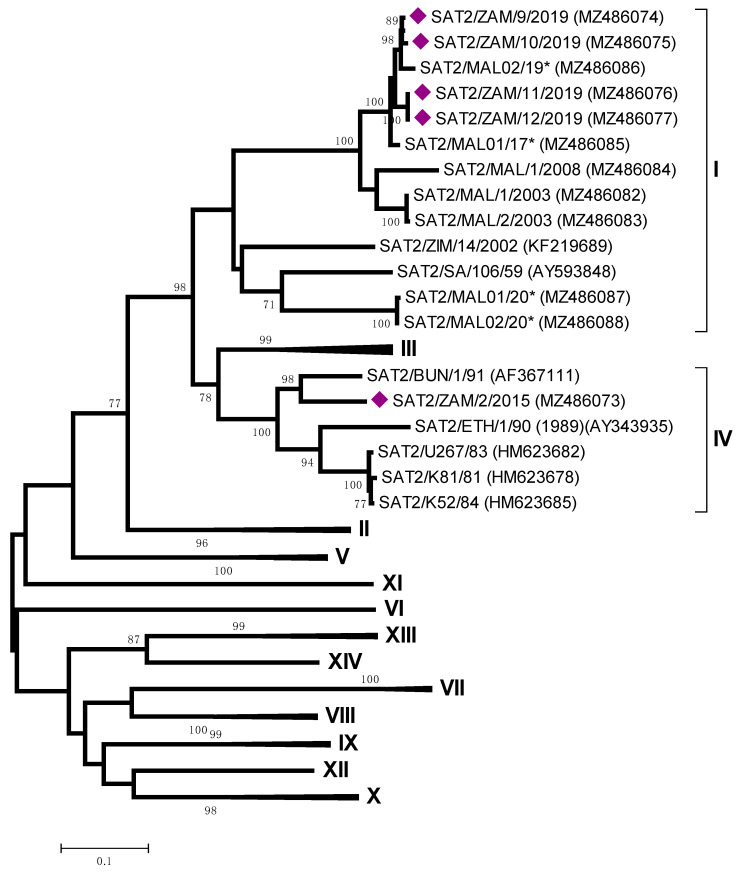
Midpoint-rooted maximum likelihood tree showing the relationships between the VP1 sequences of the 2015 and 2019 foot-and-mouth disease virus SAT-2 serotypes from Zambia (indicated with diamonds) and other contemporary and reference viruses. * Reference number not assigned by the WRLFMD.

In the Northern Province, samples from Chimula Village in Mbala (ZAM/2/2015) were serotyped as SAT-2 and clustered within topotype IV, related to viruses circulating in East Africa (Pool 4) (Figure 5). In contrast, the VP1 sequences of four additional SAT 2 viruses (ZAM/9-12/2019) from Lundazi and Vubwi Districts of Eastern Zambia were characterized as belonging to topotype I, with closest relationships with SAT2/MAL02/19 and SAT2/MAL01/17 (96.6–98.3% nt identity) collected in Malawi (Figure 5).

**Figure 6:**
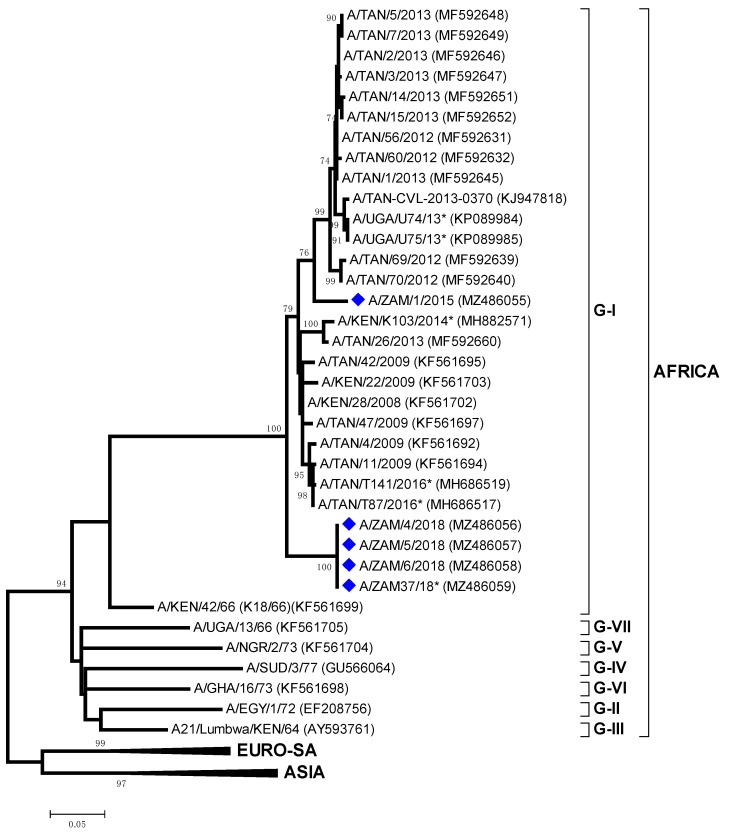
Midpoint-rooted maximum likelihood tree showing the relationships between the VP1 sequences of the 2015 and 2018 foot-and-mouth disease virus serotype A serotypes from Zambia (indicated with diamonds) and other contemporary and reference viruses. * Reference number not assigned by the WRLFMD.

This study detected five serotype A (genotype AFRICA/G-I) isolates from outbreaks that occurred in 2015 and 2018; ZAM/1/2015 from Mpulungu which was genetically distinct to four identical sequences (ZAM/4-6/2018 and A/ZAM37/18 from the Mbala, Northern Province) (Figure 6). Viruses from both of these clades were most closely related to sequences from FMDV isolates collected in Tanzania 95–96% nt identity.

## 4. Discussion

This is the first molecular epidemiology study of FMDV in Zambia undertaken to define the genetic relationships between Zambian FMD viruses and those collected from neighbouring countries. These results highlight that FMD continues to circulate in the three high-risk areas of FMD in Zambia, and also reveals notable changes in the epidemiology of FMD, including confirmed cases of FMDV serotype SAT-3 in cattle of Shangombo district of Western Province in 2015, the emergence of FMDV serotype O (O/EA-2) in Central, Southern, Copperbelt, Western and Lusaka Provinces and the introduction of the SAT-2 serotype into Eastern Zambia.

FMDV serotype SAT-3 has only been isolated previously in Zambia from wild buffalo, although infection due to this serotype was once diagnosed serologically in cattle [17,22]. This study describes the first FMDV serotype SAT-3 outbreak in domesticated cattle, believed to have been initiated by an illegal livestock movement from a neighbouring country. It has also been speculated that these outbreaks may have involved buffalo from the Sioma National Game Park after cattle were seen grazing with buffalo [36]. This national park, located in Shangombo and Sioma districts, is one of the protected areas in the Kavango Zambezi (KAZA) Transfrontier Conservation Area (TFCA), where transboundary animal movement is common and promoted especially for wildlife. As reported previously, most SAT serotype outbreaks have occurred in and around the KAZA TFCA, where approximately 1.5 million people and their livestock are resident [12,37]. Although two sequences (SAT3/BOT/P3/98 NXA-9 and SAT3/BOT/P3/98 NXA-6) collected from buffalo in north-western Botswana (Nxaraga Lagoon, Lower Boro floodplain) were clustered with the SAT3 sequences from Zambia, direct testing of the buffalo-cattle transmission hypothesis requires further sampling of buffalo populations resident in Zambia.

The first confirmed serotype O outbreak in Zambia was in Mbala District of Northern Province in 1976 [14,38]. Since then, serotype O outbreaks have only been reported in this province in three instances (1982, 2000 and 2010). This study reports sequence data for further topotype O/EA-2 outbreaks in the Northern Province and the wider circulation of this topotype into other Zambian provinces. Although these sequences represent what was considered as two primary outbreaks, their close sequence identity demonstrates that they are intimately connected, supporting suspicions that animals had been moved illegally from Northern Zambia (Mbala) into Central Zambia (Chisamba, a previously FMD-free area) in March 2018. The precise transmission routes are not known, but the closure of the abattoir in the Mbala district of Northern Zambia may have incentivized stock owners to move their animals’ southwards in search of market access. From the Chisamba district, the topotype O/EA-2 spread into other districts in Zambia where sequences have been recovered from cases in the Monze district of Southern Zambia after a disease quiescence of 11 months, when a herd of cattle were moved illegally from Chisamba [39]. FMD cases due to topotype O/EA-2 have been detected subsequently in Copperbelt and Western provinces, and further serotype O samples characterized by Ag-ELISA have been collected from Central, Lusaka and North-Western Provinces. From Monze (Southern Province), sequence data confirmed the spread of topotype O/EA-2 to Mazabuka, from where there were reports that FMD spread to the Kafue Flats, an area where animals congregate during the transhumance grazing, a common practice in Southern and Western provinces of Zambia [40,41]. There have been reports that O/EA-2 FMD infection in naïve animals is more severe with higher morbidity, a feature of these outbreaks that should be closely monitored if this topotype spreads to the other SADC states south of the Zambezi River.

The serotype SAT-2 FMDVs, identified in this study clustered within two topotypes (I and IV) (Figure 5). These two topotypes represent FMD viruses that circulate in two different endemic pools (Pool 4 for topotype IV and Pool 6 for topotype I, respectively), and these differences demonstrated that the SAT-2 outbreaks in Eastern and Northern Provinces were not epidemiologically linked. Eastern Province recorded only one previous FMD case in Egichikeni Veterinary Camp of Lundazi District in 2001 [22,42]. The FMD cases in 2019 were first reported from the Vubwi district, followed by Lundazi and lastly Chipata in May 2019. This Province shares borders with Malawi and Mozambique, and the close relationship between the outbreak viruses from Zambia and Malawi provides evidence for an epidemiological connection between FMD cases in these two countries. There is no natural barrier between the border areas of these countries, and it has been reported that livestock from the Malawian side are herded into Zambia, where they graze with Zambian livestock [43]. This outbreak was resolved following two rounds of vaccinations with a bivalent vaccine (serotypes SAT-2 and O) targeting the cattle population at risk.

For serotype A, the sequences recovered in this study represent two genetic clades within the A/AFRICA/G-I genotype, representing viruses collected from 2015 and 2018, respectively. These data suggest that there were two separate serotypes A introductions into northern Zambia from a neighbouring country in East Africa.

The data reported in this paper reflect the complex epidemiology of FMD in Zambia, where field outbreaks in domesticated species over five years have been due to four different serotypes (O, A, SAT-2 and SAT-3). The causes of these FMD outbreaks in Zambia are multifactorial, although uncontrolled livestock movement continues to play a very important role in spreading FMD viruses within the country [22,40,41]. During 2019, the potential for FMD transmission in Zambia was elevated as a consequence of a drought (November–March) [44], which resulted in a shortage of water and pasture for farmers, leading to the congregation of livestock by small ponds, a scenario which has been previously reported as contributing to the spread of infectious diseases such as FMD [21]. Furthermore, the lack of grazing pasture forced livestock keepers to sell their livestock in exchange for maize, thus facilitating livestock movement as animals moved from buyer to seller.

This paper also demonstrates the movement of FMDV serotypes and topotypes among different territories, and the importance of surveillance to rationally design vaccination campaigns in the different regions. Although presented late to assist curb the spread of FMD in Zambia, the data presented accentuates challenges faced in FMD control as previously outlined [12,15], with the time factor being predominant. Not reporting or late reporting of the disease leads to poor diagnostic ability, and furthermore makes the disease investigation devoid of the epidemiological information required for effective disease management. Increased stakeholder engagement is necessary, and should be encouraged during FMD outbreaks, as disease control proved effective with this strategy in some outbreak areas during this study. Secondly, the lack of adequate diagnostic facilities in most endemic countries like Zambia raises the argument that virus analysis in endemic settings must be locally matched with commensurate investment in FMD diagnostic infrastructure, including sample collection and processing. In addition, the use of rapid field diagnostic tests close to outbreak areas are necessary for phylogenetic analysis to have a real impact on curtailing circulating viruses during disease outbreaks. It is therefore proposed that the planned risk-based FMD control strategy for Zambia and the region takes into account the sound epidemiological assessment of the incidence and distribution of FMD provided in this paper. This includes identifying risk sources as either primary or secondary endemic eco-systems for the effective design of FMD control programs.

The introduction and circulation of novel FMD viruses in Zambia may have a bearing on the epidemiology of FMD in the country. Zambia represents an interface between endemic Pool 4 (East Africa) and Pool 6 (Southern Africa), and the spread of O/EA-2 into southern border areas in Zambia is of particular concern, as it could alter the epidemiological risks for the SADC region. Furthermore, the emergence of serotype O as a risk for SADC countries has impacted the selection, and costs associated with FMD vaccination since trivalent vaccines (containing only SAT-1, SAT-2, SAT-3 vaccine strains) are the most frequently used in the region.

## 5. Conclusions

This study describes the molecular epidemiology of FMD viruses collected from Zambia between 2015 and 2020 highlighting the recent spread of serotype O, A, SAT-2 and SAT-3 viruses in the country. These results reinforce the importance of synchronizing surveillance and disease control strategies where the emergence of new FMD virus serotypes and lineages impacts vaccine selection for the region to ensure that data is shared between countries to monitor this dynamic situation.

## Figures and Tables

**Table 1:** Details of tissue samples described in this study (2015–2020).

CVRI Ref No.	Date Collected (DD/MM/YYYY)	Species	Vet Camp/Village	District, Province	NSP	Serotype	WRLFMD Ref. No.	BVI Ref. No.	RT-qPCR	Serotype	Topotype	Lineage
MM01	02.4.2015	Bovine	Mupata Village	Mpulungu, Northern	Positive	ND	ZAM/1/2015		FMDV-GD	A	AFRICA	G-I
HS 01	28.2.2015	Bovine	Chimula Village	Mbala, Northern	Positive	ND	ZAM/2/2015	ZAM03/15	FMDV-GD	SAT 2	IV	-
Sh 01	23.10.2015	Bovine		Shangoomb, Western	Positive	ND	ZAM/3/2015	ZAM09/15	FMDV-GD	SAT 3	II (WZ)	-
LK1	18.5.2017	Bovine	Mbanga & Mulongo	Lukulu, Western	Positive	ND	ZAM/1/2017	ZAM08/17	FMDV-GD	SAT 3	II (WZ)	-
K1	04.1.2018	Bovine	Kaluwe & Siluwe	Kalabo, Western	Positive	ND						
Chisa 16-380	24.3.2018	Bovine	Chisamba	Chisamba, Central	Negative	ND	ZAM/1/2018	-	FMDV-GD	O	EA-2	-
Chisa 16-169	24.3.2018	Bovine	Chisamba	Chisamba, Central	Negative	ND	ZAM/2/2018	-	FMDV-GD	O	EA-2	-
BBR-1	24.3.2018	Bovine	Chisamba	Chisamba, Central	Negative	ND	ZAM/3/2018	-	FMDV-GD	O	EA-2	-
TS 01	24.10.2018	Bovine	Kawimbe	Mbala, Northern	Positive	ND	ZAM/4/2018	-	FMDV-GD	A	AFRICA	G-I
ES 01	24.10.2018		Kawimbe	Mbala, Northern	Positive	ND						
ES 02	24.10.2018	Bovine	Kawimbe	Mbala, Northern	Positive	ND	ZAM/5/2018	-	FMDV-GD	A	AFRICA	G-I
GS 01	24.10.2018	Bovine	Kawimbe	Mbala, Northern	Positive	ND	ZAM/6/2018	-	FMDV-GD	A	AFRICA	G-I
ES03	24.10.2018	Bovine	Kawimbe	Mbala, Northern	Positive	ND	-	ZAM37/18	FMDV-GD	A	AFRICA	G-I
VS 02	27.3.2018	Bovine	Kawimbe	Mbala, Northern	Positive	ND	-	ZAM10/18	FMDV-GD	O	EA-2	-
VS 05	27.3.2018	Bovine	Kawimbe	Mbala, Northern	Positive	ND	-	ZAM12/18	FMDV-GD	O	EA-2	-
ZRC 1	18.1.2019	Bovine	Chisamba Central	Chisamba, Central	Positive	ND	ZAM/1/2019	-	FMDV-GD	O	EA-2	-
CVRI67/19	17.7.2019			Chibombo, Central	Positive	ND						
CVRI04/19	11.2.2019	Bovine	Ufwenuka	Monze, Southern	Positive	O	ZAM/2/2019	-	FMDV-GD	O	EA-2	-
CVRI01/19	11.2.2019	Bovine	Ufwenuka	Monze, Southern	Positive	O	ZAM/3/2019	-	FMDV-GD	O	EA-2	-
CVRI08/19	11.3.2019	Bovine	Magoye	Mazabuka, Southern	Positive	O	ZAM/4/2019	-	FMDV-GD	O	EA-2	-
17562	16.3.2019			Chibombo, Central		-	ZAM/5/2019	-	NGD	-	-	-
17496	17.3.2019			Chibombo, Central		-	ZAM/6/2019	-	NGD	-	-	-
CVRI16/19	30.3.2019	Bovine	Kasako	Mazabuka, Southern	Positive	O	ZAM/7/2019	-	FMDV-GD	O	EA-2	-
CVRI15/19	30.3.2019	Bovine	Kasako	Mazabuka, Southern	Positive	O	ZAM/8/2019	-	FMDV-GD	O	EA-2	-
CVRI22/19	02.4.2019	Bovine	Mwase	Lundazi, Eastern	Positive	SAT-2	ZAM/9/2019	-	FMDV-GD	SAT 2	I	-
CVRI23/19	02.4.2019	Bovine	Mwase	Lundazi, Eastern	Positive	SAT-2	ZAM/10/2019	-	FMDV-GD	SAT 2	I	-
CVRI20/19	03.4.2019	Bovine	Zozwe & Mlawe	Vumbwi, Eastern	Positive	SAT-2	ZAM/11/2019	-	FMDV-GD	SAT 2	I	-
CVRI21/19	03.4.2019	Bovine	Mbande	Vumbwi, Eastern	Positive	SAT-2	ZAM/12/2019	-	FMDV-GD	SAT 2	I	-
CVRI25/19	03.5.2019	Bovine	Feni	Chipata, Eastern	Positive	SAT-2	-	-		SAT 2		
CVRI37/19	16.6.2019	Bovine	Lwashimba	Kapiri Mposhi, Central	Positive	O	-	ZAM06/19	FMDV-GD	O	EA-2	-
CVRI55/19	28.6.2019	Bovine	Musenga	Chingola, Copperbelt	Positive	O	-	ZAM07/19	FMDV-GD	O	EA-2	-
CVRI29/19	20.5.2019	Bovine	Chitongo	Namwala, Southern	Positive	O	-	-		O		
CVRI30/19	20.5.2019	Bovine	Chitongo	Namwala, Southern	Positive	O	-	-		O		
CVRI31/19	05.6.2019	Porcine	Hamangaba	Monze, Southern	Positive	O	-	-		O		
CVRI32/19	05.6.2019	Porcine	Hamangaba	Monze, Southern	Positive	O	-	-		O		
CVRI61/19	12.6.2019	Bovine	Sunrise (Kasupe)	Chilanga, Lusaka	Positive	O	-	-		O		
CVRI36/20	14.2.2020	Porcine	Makeni	Chilanga, Lusaka	Positive	O	-	-		O		
CVRI01/20	14.1.2020	Bovine	Kaungaleuti	Nalolo, Western	Positive	O	-	ZAM03/20	FMDV-GD	O	EA-2	-
CVRI02/20	14.1.2020	Bovine	Kaungaleuti	Nalolo, Western	Positive	O	-	-		O		
CVRI05/20	14.1.2020	Bovine	Kaungaleuti	Nalolo, Western	Positive	O	-	-		O		
CVRI06/20	14.1.2020	Bovine	Kaungaleuti	Nalolo, Western	Positive	O	-	-		O		
CVRI13/20	17.4.2020	Bovine	Luubwe	Itezhi- Tezhi, Central	Positive	O	-	-		O		
CVRI14/20	17.4.2020	Bovine	Luubwe	Itezhi- Tezhi, Central	Positive	O	-	-		O		
CVRI17/20	17.4.2020	Bovine	Luubwe	Itezhi- Tezhi, Central	Positive	O	-	-		O		
CVRI15/20	17.4.2021	Bovine	Luubwe	Itezhi- Tezhi, Central	Positive	O	-	-		O		
CVRI29/20	13.8.2020	Bovine	Makeni	Chilanga, Lusaka	Positive	O	-	-		O		
CVRI25/20	10.3.2020	Bovine	Nyawa	Kazungula, Southern	Positive	O	-	-		O		

BVI-Botswana Vaccine Institute, CVRI-Central Veterinary Research Institute, GD-Genome Detected, ND-Not done, NSP-non-structural protein (3ABC ELISA), RT-qPCR-real-time PCR, Serotype (antigen ELISA), WRLFMD-World Reference Laboratory for Foot and Mouth Disease.

## Data Availability

All nucleotide sequences generated during this study have been submitted to GenBank and have been assigned the following accession numbers: MK422556-MK422569 and MK422575-MK422608.
